# Adaptation and Evaluation of a Multi-Criteria Decision Analysis Model for Lyme Disease Prevention

**DOI:** 10.1371/journal.pone.0135171

**Published:** 2015-08-21

**Authors:** Cécile Aenishaenslin, Lise Gern, Pascal Michel, André Ravel, Valérie Hongoh, Jean-Philippe Waaub, François Milord, Denise Bélanger

**Affiliations:** 1 Research Group on Epidemiology of Zoonoses and Public Health, Faculté de médecine vétérinaire, Université de Montréal, Saint-Hyacinthe, Quebec, Canada; 2 Laboratory for Foodborne Zoonoses, Public Health Agency of Canada, Saint-Hyacinthe, Quebec, Canada; 3 Laboratoire d’Eco-Épidémiologie, Institut de Biologie, Université de Neuchâtel, Neuchâtel, Suisse; 4 Institut national de santé publique du Québec, Longueuil, Quebec, Canada; 5 Group for Research in Decision Analysis (GERAD), Montreal, Quebec, Canada; 6 Département de pathologie et microbiologie, Faculté de médecine vétérinaire, Université de Montréal, Saint-Hyacinthe, Quebec, Canada; University of Kentucky College of Medicine, UNITED STATES

## Abstract

Designing preventive programs relevant to vector-borne diseases such as Lyme disease (LD) can be complex given the need to include multiple issues and perspectives into prioritizing public health actions. A multi-criteria decision aid (MCDA) model was previously used to rank interventions for LD prevention in Quebec, Canada, where the disease is emerging. The aim of the current study was to adapt and evaluate the decision model constructed in Quebec under a different epidemiological context, in Switzerland, where LD has been endemic for the last thirty years. The model adaptation was undertaken with a group of Swiss stakeholders using a participatory approach. The PROMETHEE method was used for multi-criteria analysis. Key elements and results of the MCDA model are described and contrasted with the Quebec model. All criteria and most interventions of the MCDA model developed for LD prevention in Quebec were directly transferable to the Swiss context. Four new decision criteria were added, and the list of proposed interventions was modified. Based on the overall group ranking, interventions targeting human populations were prioritized in the Swiss model, with the top ranked action being the implementation of a large communication campaign. The addition of criteria did not significantly alter the intervention rankings, but increased the capacity of the model to discriminate between highest and lowest ranked interventions. The current study suggests that beyond the specificity of the MCDA models developed for Quebec and Switzerland, their general structure captures the fundamental and common issues that characterize the complexity of vector-borne disease prevention. These results should encourage public health organizations to adapt, use and share MCDA models as an effective and functional approach to enable the integration of multiple perspectives and considerations in the prevention and control of complex public health issues such as Lyme disease or other vector-borne and zoonotic diseases.

## Introduction

In the context of global climate changes, zoonotic and vector-borne diseases may intensify their threat to human health [1]. Lyme disease (LD) is a good example of a complex disease that is affected by global changes, including climate disruption and changing landscapes [2–5]. Transmitted to humans by a tick bite, LD is caused by the bacteria *Borrelia burgdorferi* sensu lato whose reservoirs include wild animals such as small rodents and various bird species [6]. Its main geographic distribution is Eurasia and North America. It is currently the most frequent vector-borne disease in temperate countries on Northern hemisphere with incidence rising in many of them. In Europe, about 85,000 cases are reported annually [7] and a recent adjusted estimation evaluates that the number of cases per year could reach 300,000 in the United States [8]. LD vectors have been increasingly found in new northern locations, such as in South-Eastern Canada, where *Ixodes scapularis* ticks have recently become established and where LD is emerging [9, 10].

LD preventive strategies can be divided into actions targeting human populations, such as the promotion of individual preventive behaviors through enhanced public health communications or the development of a vaccine against LD, and actions that aim to reduce the exposure of individuals by reducing tick density in the environment [11–15]. For this second category of actions, several interventions have been tested in experimental settings or in the field, and include actions that directly target tick populations via the use of acaricides or landscaping, and indirect actions that target wild animals which are hosts of the vector and/or reservoirs of the agent, such as the reduction of deer density, the treatment of deer against ticks, the treatment of small rodents against ticks and the vaccination of rodents against *Borrelia* sp (environmental preventive measures against LD are reviewed in Piesman and Eisen [11]). While most public health efforts have been focused on promoting individual preventive measures in the populations at risk in affected countries, no clear consensus has been reached among experts and public health professionals as to what constitute the best practices for LD prevention. One reason for this is that the majority of proposed strategies may have potential impacts which can be conflicting with their positive effects on human health, such as high public costs, unknown public acceptability, complex implementation and uncertain efficacy to reduce LD incidence [16].

Prevention and control of zoonoses and vector-borne diseases such as LD is a complex challenge that needs to be addressed with a systemic and transdisciplinary approach. Multi-criteria decision aid (MCDA) tools comes from the field of decision sciences and have been used to prioritize and rank public health interventions, including preventive interventions for zoonoses and vector-borne diseases [17, 16, 18]. Several multi-criteria analysis methodologies exist to study different types of problems [19], including for ranking multiple options based on a list of decision criteria, for which the PROMETHEE method (Preference Ranking Organization Method for Enrichment Evaluations) has been widely used [20, 21]. PROMETHEE method is in the family of outranking methods in MCDA and can be understood as a 10-step systematic process which can be broadly divided into two phases [16]. First, the ‘problem structuring’ phase leads to the building of a MCDA matrix that allows the evaluation of a list of possible options according to a list of decision criteria. The criteria should be exhaustive, coherent and non-reductant and should represent all important issues as identified by concerned stakeholders in relation to the problem. The measurement scale of the criteria can be either quantitative or qualitative, a flexibility that permits the incorporation of expert assessment whenever literature or field-supported data are missing on an aspect of the problem. The criteria can be weighted by different stakeholders according to the criteria’s relative importance in the decision process. Second, the ‘decision analysis’ phase proceeds to pair-wise comparisons to produce several outputs with the objective of facilitating decision-making, including stakeholder and overall group rankings of options. Option profiles and decision maps (GAIA plans [22]) are outputs that are useful for visualizing points of convergence and divergence points among stakeholders across the range of issues captured by the identified criteria.

Aenishaenslin and colleagues [16] used MCDA to prioritize actions for LD surveillance, prevention and control in the province of Quebec, Canada, in an epidemiological context of LD emergence with low global incidence. One of the main conclusions made by the authors was that a part of the models parameters, and particularly the list of criteria, could be seen as a generic structure for other vector-borne and zoonotic disease management contexts.

Based on this hypothesis, the aim of the current study was to evaluate the applicability of the MCDA model for LD preventive interventions previously developed in Quebec in a different epidemiological context. More precisely, we had the objective to identify parameters that were directly transferable and those that needed to be modified to capture issues specific to the new context. For this purpose, Switzerland was chosen as a result of its contrasting LD epidemiological situation. In this country, LD has been endemic for over thirty years with a high incidence of 83 cases per 100,000 inhabitants estimated in 2010 [23]. LD ecology differs between Europe and North America with a greater diversity of species that can be reservoirs of the bacteria, hosts species of the vector and the primary tick vector, *Ixodes ricinus*, generally found in European regions [24]. Despite this, the majority of the recommended preventive strategies are believed to be applicable in both contexts. A secondary objective of the study was to contrast the main results produced with the Swiss MCDA model with those of the Quebec MCDA model [16].

## Materials and Methods

### Adaptation of the MCDA model for the Swiss context

As per Aenishaenslin and al. (2013), a MCDA model was developed to prioritize LD preventive interventions in the province of Québec, Canada, as part of a larger project leading to the development of several MCDA models related to LD management [16]. This Quebec MCDA model included a comprehensive list of 12 criteria spread across four general categories (public health, animal and environmental health, social impacts and economic, strategic and operational impacts) that were weighted by eight stakeholders (experts from academic and public organisations). Sixteen preventive interventions were proposed targeting either human or tick populations and evaluated over all identified criteria to produce a performance matrix (or MCDA matrix). Multi-criteria analysis (PROMETHEE method) produced a number of results, including a group ranking of the prioritized interventions [16].

For the current study, a participatory process was undertaken with a group of Swiss stakeholders. Members of the National Reference Centre for Tick-Borne Diseases were invited to participate in the process [25]. At the time of the study, this center was mandated to provide recommendations for LD and other tick-borne diseases prevention in Switzerland and its members included representatives of governmental, academic and civic organisations concerned by these issues. Nine members participated in the study (Table 1). Informed written consent was obtained for all participants. This procedure and the study protocol was reviewed and approved by the Ethical Committee for Health Research of the University of Montreal (CERES).

**Table 1:** Composition of the Swiss stakeholder group.

Organisations (number of stakeholders)
Swiss federal office of Public Health (1)
Swiss federal veterinary office (1)
Diagnostic Laboratory Experts (2)
Neuchâtel canton medical officer (1)
Swiss National Accident Insurance Fund representative (1)
Lyme disease patient advocacy group representative (1)
Academic experts (2)

The participating stakeholders were asked to act as the voice of their organisation, and invited to consult with other members of their organisation in order to validate the list of criteria, their measurement scales, and the list of interventions to be included in the model as well as their criteria weighting scheme. A focus group and individual follow-up interviews with those unable to attend the initial focus group (n = 2) were conducted between May and August 2012 to collect their input. Criteria weighting was performed by each stakeholder by distributing 100 points among the finalized list of criteria via an Excel spreadsheet tool (Microsoft Excel software version 2007). Stakeholders had the choice of two weighting strategies: they were asked to either divide 100 points among the 16 criteria, or to divide 100 points among the four main categories of criteria, and then to divide the points among the sub-criteria. The tool developed to perform the weighting automatically calculated the weights by category of criteria, and the weights for each individual criterion, as the stakeholders completed the calculation sheet. They were asked to verify whether their final weights per category and per individual criteria truly represented their perceived relative importance.

The evaluation of the performance of proposed interventions over all criteria was done based on a modified version of the Quebec dataset, adapted to the Swiss context. This step consisted of evaluating the performance score of each intervention for each criterion according to its measurement scale. Data from the literature were updated and new data were incorporated for modified interventions and criteria. Evaluations that needed contextual information, such as the level of complexity in the implementation of an intervention and the impact on an organisation’s credibility, were validated and/or modified by the stakeholder group.

### Multi-criteria analysis

Multi-criteria analysis were conducted using D-Sight Software (version 4.2.0), using the PROMETHEE method [26, 20]. The PROMETHEE method performs pair-wise comparisons of each intervention’s performance based on a preference function in order to compute a net outranking flow, referred to as ‘scores’ in this study, that enables group rankings of a list of interventions. Algorithms used to calculate these scores are described in Brans et al. (1986), and a review of PROMETHEE method applications can be found in Behzadian and colleagues [21]. PROMETHEE method also enables to perform individual rankings for each stakeholder. This can help each stakeholder to better understand his/her position and preferences with regard to the group, and can be an interesting tool to negotiate with other stakeholders regarding a decision. Individual rankings are not presented in this paper.

For this study, group rankings were computed under two different scenarios: the first consisting of all criteria defined by the Swiss stakeholders (scenario A), and the second consisting only of the criteria identified in the original Quebec model (scenario B). The complete modified list of interventions was used for both scenarios. For scenario B, Swiss stakeholder’s weights were normalized to total 100 points in order to keep the size of the relative importance of the remaining criteria. This was done to improve the comparison between countries. Group rankings were computed using specific algorithms based on a pair wise method of analysis to calculate scores for each intervention according to its performance on all criteria and on all stakeholders weighting scheme [27]. Intervention scores represent the relative performance of an intervention with respect to another, and as such scores do not have individual meanings by themselves. Analyses were conducted using a preference function with a linear scale for all criteria.

A GAIA (Geometrical Analysis for Interactive Aid) decision map was produced to visually assess the level of agreement between stakeholders under scenario A. GAIA decision maps are graphical tools of the PROMETHEE method that can be generated by the D-Sight Software [27, 22]. They are two-dimensional representation of a stakeholder’s position around a decision axis (in red) representing the direction of the ‘best’ interventions (positioned around this axis), based on the evaluation of intervention performances over all criteria combined with stakeholders group weighting schemes. The proximity of stakeholder’s positions to one another indicates proximity in their overall preferences for specific decision criteria. This graphical representation can thus be used to identify convergence and divergence (in other words, the level of agreement) between stakeholders. Results were discussed with Swiss stakeholders during a second focus group conducted in November 2014 to validate the representativeness of the final model in the Swiss context. Eight out of the original nine stakeholders participated.

### Comparative analysis

Three elements of the decision model were specifically selected to evaluate the adaptability of the model from the Quebec to the Swiss context: 1) the list of identified criteria and 2) the list of selected interventions, and 3) the group rankings in scenario A and B. The group ranking in both scenarios were compared qualitatively to assess whether criteria added in the Swiss model had a significant impact on the resulting group ranking or not. Stakeholder weighting schemes between the two countries were also compared using normalized weights under scenario B (only Quebec model criteria) for the Swiss stakeholders. Overall group rankings were also compared between country models.

## Results

The 12 original criteria from the Quebec model were considered as important and were retained in the model without modification. In addition, the Swiss stakeholders suggested four new criteria, for a total of 16 criteria. The efficacy of the intervention to reduce the incidence of disseminated LD cases was added to the ‘public health’ category of criteria (disseminated LD cases represents the subset of cases for which the infection has become systemic, resulting generally in a more severe illness). The ability of the intervention to raise the level of public awareness was added to the ‘social impacts’ category. The sustainability of the intervention’s effect and the level of coherence within the European strategy to prevent LD were added to the ‘economic, operational and strategic’ category (Table 2). The description of measurement scales for each criterion can be found in S1 Table.

**Table 2:** Original and new criteria included in the Swiss model.

Category	Original criteria from the Quebec model	New criteria added in the Swiss model
Public health criteria (PHC)	PHC1 Reduction in incidence of human cases	PHC4 Reduction in incidence of disseminated LD human cases
	PHC2 Reduction in entomological risk	
	PHC3 Impacts of adverse health effects	
Animal and environmental health criteria (AEC)	AEC 1 Impact on habitat	None
	AEC 2 Impact on wildlife	
Social impact criteria (SIC)	SIC 1 Level of public acceptance	SIC3 Level of public awareness
	SIC 2 Proportion of population benefitting from intervention	
Strategic, economic and operational impact criteria (SEC)	SEC1 Cost to the public sector	SEC6 Sustainability of effect
	SEC2 Cost to the private sector	SEC7 Level of coherence with the European strategies
	SEC3 Delay before results	
	SEC4 Complexity	
	SEC5 Impact on organisation’s credibility	

The list of potential interventions was modified following discussion with stakeholders. Six interventions were removed, two were modified and two new interventions were added, resulting in a final list of 13 interventions labeled INT0 to INT12 (see Table 3: modification details and justifications). The resulting performance matrix can be found in S2 Table. During the second focus group meeting held in November 2014, all stakeholders agreed that the modified model (criteria, interventions and performance matrix) was representative of important issues relative to LD prevention in Switzerland.

**Table 3:** Swiss modifications made to the original list of proposed interventions.

Category	Original interventions	Modification and justification	Modified list of interventions
Human targeted	Status quo, i.e. basic preventive communication on LD risk through official websites.	Kept in the model.	INT0 Status quo
	Excluding people from high-risk public areas	Modified for: Reduction of human visits to high-risk public areas via the use of fences or prohibitive signs. High level of concern of public acceptance in case of formal interdiction. Complete exclusion from high-risk areas may be difficult given that LD is endemic in all Swiss territory under 1500 m asl.	INT1 Reduction of human visits to high-risk public areas via the use of fences or prohibitive signs
	Human vaccination [28]	Kept in the model. Not currently available, but a possible future option.	INT2 Human vaccination
	-	Addition of: Large communication campaign [29, 12]. A large communication campaign would be interesting in Switzerland given that all territory is considered as endemic for LD under 1500 m asl.	INT3 Large communication campaign
	Making available special Lyme disease diagnostic/treatment clinic(s)	Modified in two different interventions: Making available special clinics for diagnosis of complex cases: Improve laboratory diagnostic of complex LD cases; Making available special clinics for complex LD cases management: Improve management of complex LD cases. One main concern in Switzerland was to reduce the incidence of complex LD cases such as neuroborreliosis.	INT4 Making available special clinics for diagnosis of complex cases; INT5 Making available special clinics for complex LD cases management
	-	Addition of: Learning sessions for physicians. One main concern in Switzerland was to reduce the incidence of complex LD cases such as neuroborreliosis. Enhancing physician competencies may improve LD diagnosis, reduce the incidence of complex LD cases and strengthen the management of complex LD cases.	INT6 Learning sessions for physicians
Vectors targeted through environmental interventions	Small scale acaricide application to kill free-living ticks [13]	Kept in the model.	INT7 Small scale acaricide application
	Large scale acaricide application to kill free-living ticks	Removed. Large-scale interventions should not be considered for the Swiss context given the limited superficies of wooded areas in the country, and the great value put on the protection of the environment.	-
	Small scale landscaping (removal of tick habitats) [14, 30]	Kept in the model.	INT8 Small scale landscaping
	Large scale Landscaping (removal of tick habitats)	Removed. Large-scale interventions should not be considered for the Swiss context given the limited superficies of wooded areas in the country, and the great value put on the protection of the environment.	-
	Application of desiccants / insecticidal soap	Removed. This intervention was considered as non-applicable in Switzerland, given the great value put on the protection of the environment.	-
Vectors targeted through hosts interventions	Installation of devices for topical acaricide application to deer (‘4-poster' device) [31, 32]	Kept in the model.	INT9‘4-poster' device
	Feed-administered ivermectin to deer at bait stations to control ticks	Removed. The ‘4-poster’device was considered as a better option for targeting deer.	-
	Deer hunting [33]	Kept in the model.	INT10 Deer hunting
	Deer culling	Removed. High level of concern of public acceptance.	-
	Bait boxes to deliver a passive application of fipronil to rodents (‘Damminix’ devices)[34]	Removed. The ‘Damminix’ device was considered as a better option for targeting rodents.	-
	Exclusion of deer by fencing [35]	Kept in the model.	INT11 Exclusion of deer by fencing
	‘Damminix’ device [36]	Kept in the model.	INT12 ‘Damminix’ device

**Fig 1:**
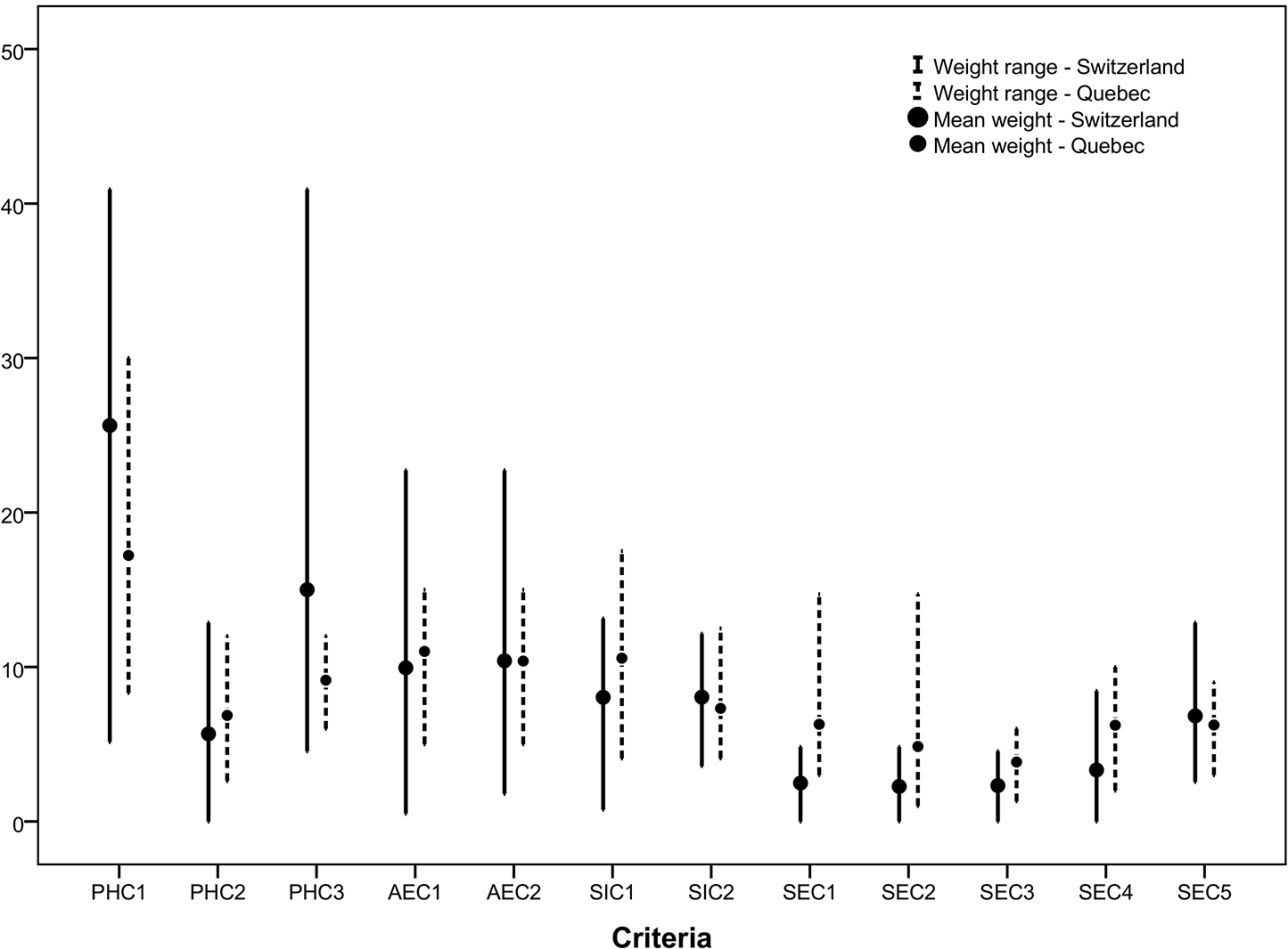
Distribution of weights for Swiss (dark lines. n = 9) and Quebec (dotted lines. n = 8) stakeholders for the 12 original criteria. Legend: PHC1 Reduction in incidence of human cases; PHC2 Reduction in entomological risk; PHC3 Impacts of adverse health effects; AEC1 Impact on habitat; AEC2 Impact on wildlife; SIC1 Level of public acceptance; SIC2 Proportion of population benefitting from intervention; SEC1 Cost to the public sector; SEC2 Cost to the private sector; SEC3 Delay before results; SEC4 Complexity; SEC5 Impact on organisation’s credibility.

Weights per category and specific criteria varied according to stakeholders (Table 4). For 7 out of 9 stakeholders, the highest weight was given to the public health category, but other category weights varied greatly between stakeholders. Fig 1 shows the distribution of criteria weights for Swiss and Quebec stakeholders from the Quebec model for scenario B (showing only criteria included in the Quebec model). Fig 1 highlights the variation in weight distribution between Swiss and Quebec stakeholders, and shows how for seven criteria out of twelve, the weight range was larger among Swiss stakeholders. The largest variation can be observed for criteria ‘Adverse public health effect’ (PH3), where Swiss stakeholders weights ranged from 3 to 40, compared to a weight range of only 6 to 12 by Quebec stakeholders [16].

**Table 4:** Stakeholder weights by category and for criteria.

		S1	S2	S3	S4	S5	S6	S7	S8	S9
Category weights	Public health criteria (PHC)	33	48	35	80	59.8	40.0	25	50	40
	Animal and environmental health criteria (AEC)	16	20	20	5	2	20	25	10	10
	Social impact criteria (SIC)	18	23.2	25	5	20.6	26	25	15	20
	Strategic, economic and operational impact criteria (SEC)	33	8.8	20	10	17.7	14	25	25	30
Individual criteria weights	PHC1 Reduction in incidence of human cases	16	30	3.5	40	15	10	17.5	25	14
	PHC2 Reduction in entomological risk	6.6	5	8.8	0	0.6	10	0	2.5	4
	PHC3 Impacts of adverse health effects	5	10	8.8	40	9	10	2.5	12.5	8
	PHC4 Reduction in incidence of disseminated LD human cases	5	3	14	0	35.3	10	5	10.0	14
	AEC1 Impact on habitat	8	10	10	0.5	1	10	12.5	5.0	5
	AEC2 Impact on wildlife	8	10	10	4.5	1	10	12.5	5.0	5
	SIC 1 Level of public acceptance	4.5	6.8	7.5	0.8	7.2	9	2.5	6.0	6
	SIC 2 Proportion of population benefitting from intervention	9	4.4	7.5	3.5	6.2	9	2.5	3.0	6
	SIC3 Level of public awareness	4.5	12	10	0.8	7.2	8	20	6.0	8
	SEC1Cost to the public sector	1.7	0.5	3	1.5	2.6	2	0	1.3	3
	SEC2 Cost to the private sector	1.7	0	2.4	1	2.6	2	0	1.3	3
	SEC3 Delay before results	1.7	0.5	1	0	0.9	2	2.5	2.5	3
	SEC4 Complexity	5	0.5	0.6	3	1.8	2	0	6.3	3
	SEC5 Impact on organisation’s credibility	6.6	6.8	5	3	7.1	2	2.5	3.8	6
	SEC6 Sustainability of effect	9.9	0.5	6	1	1.8	2	17.5	6.3	6
	SEC7 Level of coherence with the European strategies	6.6	0	2	0.5	0.9	2	2.5	3.8	6

**Fig 2:**
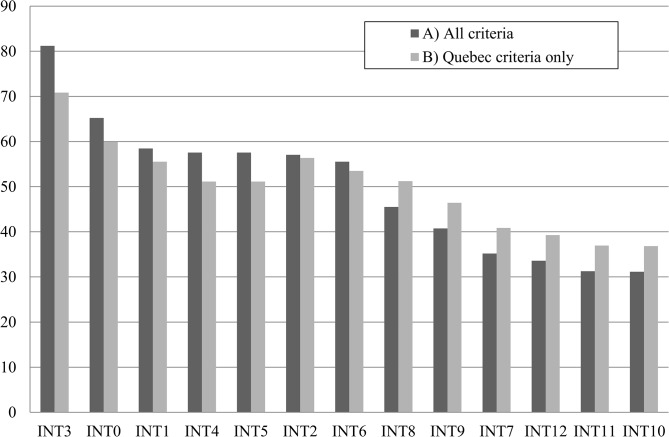
Effect of Swiss criteria removal on intervention scores (Y axis). Legend: In scenario (A), the overall group ranking considered all criteria from the Swiss model and in scenario (B). The overall group ranking considered only the original criteria from the Quebec model. The model becomes less discriminating between the “best” and “worst” interventions when Swiss criteria are removed (INT0 Status quo; INT1 Reduction of human visits to high-risk public areas via the use of fences or prohibitive signs; INT2 Human vaccination; INT3 Large communication campaign; INT4 Making available special clinics for diagnosis of complex cases; INT5 Making available special clinics for complex LD cases management; INT6 Learning sessions for physicians; INT7 Small scale acaricide application; INT8 Small scale landscaping; INT9‘4-poster' device; INT10 Deer hunting; INT11 Exclusion of deer by fencing; INT12 ‘Damminix’ device).

The overall group ranking performed under scenario A (all criteria) shows that one intervention clearly outperforms all the others: ‘Large communication campaign’ (INT3), as the preferred intervention, with a score of 81, followed by ‘Status quo’ (INT0) (score = 65) and ‘Reduction of human visits to high-risk public areas via the use of fences or prohibitive signs’ (INT1) (score = 58.5) (Table 5). All interventions targeting human populations (INT0 to INT6) were positioned in a higher rank than interventions from other categories (INT7 to INT12, targeting ticks through direct or indirect actions) (Table 5). The first top two ranked interventions, INT3 and INT0, were the same under both scenarios A and B (Table 5, Fig 2). When ranks were compared under scenarios A and B, we observed that the most important differences in rank orderings were found for interventions ‘Special clinics for diagnosis of complex cases’ (INT4) and ‘Special clinics for complex cases management’ (INT5), which moved from position 4 in scenario A to position 7 in scenario B (Table 5, Fig 2A and 2B). ‘Human vaccination’ (INT2) gained two positions and moved from position 5 in scenario A to position 3 in scenario B. All other interventions changed by no more than one position in the order of ranking.

**Table 5:** Group ranking of interventions under scenario A (all criteria) and B (Swiss criteria removed and weights normalized for all stakeholders).

	Scenario A		Scenario B	
	Considering all criteria		Considering only Quebec model criteria	
Interventions	Rank	Score	Rank	Score
INT3 Large communication campaign	1	81	1	71
INT0 Status quo	2	65	2	60
INT1 Reduction of human visits to high-risk public areas	3	58.5	4	55.5
INT4 Making available special clinics for diagnosis of complex cases	4	57.5	7	51
INT5 Making available special clinics for complex LD cases management	4	57.5	7	51
INT2 Human vaccination	5	57	3	56.5
INT6 Learning sessions for physicians	6	55.5	5	53.5
INT8 Small scale landscaping	7	45.5	6	51
INT9‘4-poster' device	8	40.5	9	46.5
INT7 INT7 Small scale acaricide application	9	35	8	41
INT12‘Damminix’ device	10	33.5	12	39.5
INT11 Exclusion of deer by fencing	11	31.5	10	37
INT10 Deer hunting	12	31	11	37

Note: Scores showed in this table are transformed net flows produced by PROMETHEE method as produced by D-sight software [27]

One second observation is that the range between maximum and minimum scores of interventions decreases from scenario A (maximum score = 81 and minimum score = 31) to scenario B (maximum score = 71 and minimum score = 37). The score of an intervention calculated with the PROMETHEE method can be considered as a numerical representation of ‘how well’ an intervention outranks another, considering its intrinsic performance on each criterion and all stakeholders weighting schemes. Globally, higher an intervention scores when compared to other intervention’s scores, more performant it is relative to the others. On the contrary, if all intervention scores are closed to each other, it is harder to clearly identify interventions that truly outrank others. Thus, the score range difference suggests that preference for the best-ranked interventions was stronger under the first scenario, when all criteria were taken into account (Table 5).

The GAIA decision map produced under scenario A showed a good level of agreement among stakeholders, with all stakeholders (S1 to S9) positioned in the same general direction as the overall group decision vector shown in red (S1A Fig). Interventions targeting ticks through direct actions on the environment (INT7 and INT8, in green in the map) or through actions on hosts (INT9 to INT12, in red in the map) shown on the left side of the decision map suggest that these interventions were not preferred by the group. This was also the case in the analysis performed with the Quebec model (S1B Fig).

## Discussion

Criteria and their general category from the Quebec model were directly transposable to the Swiss context. The addition of four new criteria and follow-up discussion with Swiss stakeholders suggests that the new model succeeded in capturing specific issues of the LD epidemiological situation and geopolitical context of Switzerland. The inclusion of ‘Reduction in incidence of disseminated LD cases’ (PHC4) is revealing. By including this criterion that focuses on the reduction of a subset of LD cases in addition to ‘Reduction of LD incidence’ (PHC1), Swiss stakeholders underlined their specific concerns relating to severe cases. Notably, two stakeholders (S3 and S5) put more weights on this criterion than on PHC1. Given that early LD cases can be treated most often successfully, it is understandable that a high-incidence region may want to prioritize actions to reduce severe LD cases which are of particular concern for the population and which may have the higher societal costs, in contrast to a lower incidence region such as the province of Quebec. The addition of ‘Level of public awareness’ (SIC3) and ‘Sustainability of effect’ (SEC6) can also be understood in light of the country’s LD history: the Swiss population has coped with this ongoing public health issue for a long time and may have observed or perceived that short-term actions may not be appropriate in their context. Finally, the inclusion of ‘Level of coherence with European strategies’ (SEC7) is understandable given the geographical and political situation of Switzerland, even if only low weights were attributed to this criterion by all stakeholders (weights range from 0 to 7).

The proposed interventions listed in the Quebec model needed modifications to suit the Swiss situation and this may be explained by the geographical and the social context of Switzerland. For example, as stated by stakeholders during the focus groups, the Swiss population has placed great value on sustainability and on the protection of its environment. Consequently, all large scale environmental actions, short-term interventions and those with little literature support on efficacy were removed.

The larger weight ranges among Swiss stakeholders compared to the Quebec study may be explained by examining the composition of the stakeholder groups. In Switzerland, an advisor group on tick-borne diseases already exists within the National Reference Centre for Tick-Borne Diseases [25]. This group includes a representative of the LD patients advocacy group in Switzerland, as well as a representative of the Swiss Accident Insurance Fund, which is responsible for insurance coverage of LD patients in Switzerland, and hence was more heterogeneous in terms of perspectives and concerns than the group that had participated in the Quebec study, which was mostly composed of public health and environmental specialists working in public organisations or academic institutions. Keeping this observation in mind, it is indeed interesting to see that a good level of agreement (comparable with the Quebec study) was still achieved. The addition of the four criteria in the Swiss context had the effect of making the model more ‘discriminating’ between interventions, by increasing the distance between scores of the highest and lowest ranked interventions (Fig 2). This highlights the added value of the validation process with local stakeholders.

Finally, the fact that the top ranked interventions are those targeting human populations (top six ranked interventions in Switzerland) was also observed in the Quebec study (four on the five top ranked interventions) [16]. In this previous study, the implementation of large communication campaigns was not included as an intervention, so we cannot directly compare the best-ranked interventions. One important point to keep in mind when using MCDA is that one strength of the approach is not to identify one unique or best solution, but rather to identify complementary interventions. This can be done using the interventions profiles, which are not presented in this paper, but which are described in [16].

This study focused on the evaluation of an existing MCDA model developed in Quebec for the Swiss context and we see a few key limitations to the approach used. A list of criteria and interventions from the Quebec model were presented and adjusted by Swiss stakeholders, but these criteria might have been different if Swiss stakeholders had created their own model, without reference to the Quebec model. This method was used in accordance to our study objective, but has likely influenced Swiss stakeholders in the inclusion of or in the choice of a measurement unit for certain issues relative to the decision-making process.

An important consideration in designing MCDA models is that criteria should be independent, in order to avoid double counting when calculating global scores if two criteria measure the same impacts. Some criteria identified in the Swiss model are interrelated. For example, the reduction of the entomological risk, the reduction of Lyme disease cases and the reduction of disseminated LD cases are not fully independent. They represent three main objectives of LD prevention and are indicators of the effectiveness of the intervention. To include the three of them may have led to inflate the importance of this effectiveness consideration in the global rankings and is a limitation of the current study. Nevertheless, weighting the criteria by taking into account the general category can prevent this bias by assuring that the weight of each category of issues truly represents its perceived importance. Full independence of criteria is hard to achieve when dealing with complex problematics such as LD prevention. Moreover, eliminating criteria that are perceived by stakeholders as important in the decision-making process because they are not completely independent can decrease the representability of real issues and limit the use of the model by decision-makers. Also, the list of criteria in this study was developed in order to achieve a balance between sufficient independence and comprehensiveness to capture LD prevention issues in Switzerland.

Another limit to consider in the interpretation of the results is the use of a normalization process to calculate weights of the stakeholders when removing the added criteria. These weights were used to allow comparisons between Quebec and Swiss stakeholders in Fig 1, and to perform group ranking under scenario B. This methodological choice may have introduced some bias in the weighting schemes, as stakeholders might have preferred to change the distribution of the remaining weights if the list of criteria was reduced. The impact of this bias is probably minimal.

Also, performance evaluations of interventions used qualitative categorical indicators for every criterion. If ordinal scales are well suited for the evaluation of certain types of criteria, such as the level of complexity, numerical scales would be more appropriate for other criteria, such as the level of reduction in human case incidence and the costs for the public and the private sectors. This was also the case in the Quebec model and reflects the availability of data at the time of the study. In the other hand, the fact that MCDA models can include qualitative evaluations such as expert assessments can also be considered as a strength of this approach, because it allows to take into account issues for which data are not available or not representative of the context, instead of completely ignoring them or their limits. Moreover, it can help to identify the main knowledge gaps and orient future researches.

Finally, both the Quebec and the Swiss models used the PROMETHEE method and D-Sight Software to perform multi-criteria analysis, which is desirable in order to compare the results. However, other methods and software for MCDA exist [21, 37]. Guidelines have been proposed to help select an appropriate MCDA method [38], but the impact of the use of different MCDA methods and /or software on one particular prioritization problematic in the field of public health has not been well described in the published literature.

## Conclusion

The current study suggests that beyond the specificity of the MCDA models developed for Quebec and adapted for Switzerland, their general structure captures the fundamental and common issues that characterize the complexity of vector-borne disease prevention, as all criteria and most of the interventions were directly transferable from one context to the other. Nonetheless, the model adaptation process, performed in this study using a participatory approach with local stakeholders, appears important to enhance the specificity of the model to local considerations.

Moreover, the generic parameters of the MCDA models developed in Quebec and in Switzerland, and particularly the general categories of criteria, are well aligned with the ‘One health’ approach, which encourages international, interdisciplinary and cross-sectoral actions for diseases at the human-animal-environment interface [39]. We believe that the generic structure of MCDA models developed in Quebec and adapted in Switzerland could provide a practical tool for the management of vector-borne and zoonotic diseases in coherence with the ‘One health’ perspective.

These results should encourage public health organizations to adapt, use and share MCDA models as an effective and functional approach to enable the integration of multiple perspectives and considerations in the prevention and control of complex public health issues such as Lyme disease or other vector-borne and zoonotic diseases. In light of these findings, we believe that further studies would be of great value to enhance our understanding on the notion of a core set of criteria that would be widely applicable to framing the relative efficacy of possible public health interventions for vector-borne and other zoonotic diseases.

## Supporting Information

S1 Fig GAIA decision maps for (A) the 9 Swiss stakeholders (S1 to S9) considering all criteria, interventions and weighting schemes (Delta = 95.1%, meaning that 95.1% of the information is conserved in the two-dimensional figure) and (B) the 8 Quebec stakeholders (S1 to S8) as computed in Aenishaenslin et Al. 2013 (16). (PDF)

S1 Table Measurement units for all selected criteria. (DOCX)

S2 Table Performance matrix of the model adapted for Switzerland. (DOCX)

## References

[1] Gage KL, Burkot TR, Eisen RJ, Hayes EB. Climate and vectorborne diseases. Am J Prev Med. 2008 Nov;35(5):436–50. 10.1016/j.amepre.2008.08.030 18929970

[2] Brownstein JS, Holford TR, Fish D. Effect of climate change on Lyme disease risk in North America. Ecohealth. 2005 3;2(1):38–46. 19008966 10.1007/s10393-004-0139-x PMC2582486

[3] Randolph SE. Evidence that climate change has caused 'emergence' of tick-borne diseases in Europe? Int J Med Microbiol. 2004 4;293 Suppl 37:5–15. 15146980 10.1016/s1433-1128(04)80004-4

[4] Randolph SE. The shifting landscape of tick-borne zoonoses: tick-borne encephalitis and Lyme borreliosis in Europe. Philos Trans R Soc Lond B Biol Sci. 2001 7 29;356(1411):1045–56. 11516382 10.1098/rstb.2001.0893 PMC1088499

[5] Brownstein JS, Skelly DK, Holford TR, Fish D. Forest fragmentation predicts local scale heterogeneity of Lyme disease risk. Oecologia. 2005 12;146(3):469–75. 16187106 10.1007/s00442-005-0251-9

[6] Stanek G, Wormser GP, Gray J, Strle F. Lyme borreliosis. Lancet. 2012 2 4;379(9814):461–73. 10.1016/S0140-6736(11)60103-7 21903253

[7] WHO. Lyme borrelioses in Europe: influences of climate and climate change, epidemiology, ecology and adaptation measures Denmark: World Health Organisation; 2006.

[8] CDC. CDC provides estimate of Americans diagnosed with Lyme disease each year. 2013; Available: http://www.cdc.gov/media/releases/2013/p0819-lyme-disease.html.

[9] Ogden NH, Artsob H, Lindsay LR, Sockett PN. Lyme disease: a zoonotic disease of increasing importance to Canadians. Can Fam Physician. 2008 10;54(10):1381–4. 18854461 PMC2567255

[10] Ogden NH, Lindsay LR, Morshed M, Sockett PN, Artsob H. The rising challenge of Lyme borreliosis in Canada. Can Commun Dis Rep. 2008 1;34(1):1–19. 18290267

[11] Piesman J, Eisen L. Prevention of tick-borne diseases. Annu Rev Entomol. 2008;53:323–43. 17877457 10.1146/annurev.ento.53.103106.093429

[12] Mowbray F, Amlot R, Rubin GJ. Ticking all the boxes? A systematic review of education and communication interventions to prevent tick-borne disease. Vector Borne Zoonotic Dis. 2012 9;12(9):817–25. 10.1089/vbz.2011.0774 22607072 PMC3438805

[13] Piesman J. Strategies for reducing the risk of Lyme borreliosis in North America. Int J Med Microbiol. 2006 5;296 Suppl 40:17–22. 16524769 10.1016/j.ijmm.2005.11.007

[14] Poland GA. Prevention of Lyme disease: a review of the evidence. Mayo Clin Proc. 2001 7;76(7):713–24. 11444404 10.4065/76.7.713

[15] Poland GA, Jacobson RM. The prevention of Lyme disease with vaccine. Vaccine. 2001 3 21;19(17–19):2303–8. 11257352 10.1016/s0264-410x(00)00520-x

[16] Aenishaenslin C, Hongoh V, Cisse HD, Hoen AG, Samoura K, Michel P, et al Multi-criteria decision analysis as an innovative approach to managing zoonoses: results from a study on Lyme disease in Canada. BMC Public Health. 2013;13:897 10.1186/1471-2458-13-897 24079303 PMC3850527

[17] Del Rio Vilas VJ, Burgeno A, Montibeller G, Clavijo A, Vigilato MA, Cosivi O. Prioritization of capacities for the elimination of dog-mediated human rabies in the Americas: building the framework. Pathog Glob Health. 2013 10;107(7):340–5. 24392676 10.1179/2047773213Y.0000000122 PMC4083153

[18] Marsh K, Dolan P, Kempster J, Lugon M. Prioritizing investments in public health: a multi-criteria decision analysis. J Public Health (Oxf). 2013 9;35(3):460–6. 23241415 10.1093/pubmed/fds099

[19] Roy B. Méthodologie multicritère d'aide à la décision Paris: Economica; 1985.

[20] Brans JP, Vincke P, Mareschal B. How to select and how to rank projects: The Promethee method. Eur J Oper Res. 1986;24(2):228–38.

[21] Behzadian M, Kazemadeh RB, Albadvi A, Aghdasi M. PROMETHEE: A comprehensive literature review on methodologies and applications. Eur J Oper Res. 2010 1 1;200(1):198–215.

[22] Mareschal B, Brans J-P. Geometrical representations for MCDA. Eur J Oper Res. 1988;34(1):69–77.

[23] Swiss Federal Public Health Office. La borréliose de Lyme: Enquête Sentinella 2008 à 2010. Bulletin de l'Office fédéral de la santé publique. 2011;17 Octobre 2011(42):895–8.

[24] Piesman J, Gern L. Lyme borreliosis in Europe and North America. Parasitology. 2004;129 Suppl:S191–220. 15938512 10.1017/s0031182003004694

[25] National Reference Centre for Tick-Borne Diseases. Available: http://www2.unine.ch/cnrt/page-11421.html.

[26] Brans JP, Mareschal B. The PROMETHEE GAIA decision support system for multicriteria investigations. Investigation Operative. 1994;4(2):107–17.

[27] Hayez Q, De Smet Y, Bonney J. D-Sight: A new decision making software to address multi-criteria problems. IGI Global; 2012 p. 1–23.

[28] Shen AK, Mead PS, Beard CB. The Lyme disease vaccine—a public health perspective. Clin Infect Dis. 2011 2;52 Suppl 3:s247–52. 10.1093/cid/ciq115 21217171

[29] Daltroy LH, Phillips C, Lew R, Wright E, Shadick NA, Liang MH. A controlled trial of a novel primary prevention program for Lyme disease and other tick-borne illnesses. Health Educ Behav. 2007 6;34(3):531–42. 17468463 10.1177/1090198106294646

[30] Brei B, Brownstein JS, George JE, Pound JM, Miller JA, Daniels TJ, et al Evaluation of the United States Department of Agriculture northeast area-wide tick control project by meta-analysis. Vector Borne Zoonotic Dis. 2009 Aug;9(4):423–30. 10.1089/vbz.2008.0150 19650737 PMC2904192

[31] Solberg VB, Miller JA, Hadfield T, Burge R, Schech JM, Pound JM. Control of Ixodes scapularis (Acari: Ixodidae) with topical self-application of permethrin by white-tailed deer inhabiting NASA, Beltsville, Maryland. J Vector Ecol. 2003 Jun;28(1):117–34. 12831136

[32] Schulze TL, Jordan RA, Hung RW, Schulze CJ. Effectiveness of the 4-Poster passive topical treatment device in the control of Ixodes scapularis and Amblyomma americanum (Acari: Ixodidae) in New Jersey. Vector Borne Zoonotic Dis. 2009 8;9(4):389–400. 10.1089/vbz.2008.0160 19650733

[33] Deblinger RD, Wilson ML, Rimmer DW, Spielman A. Reduced abundance of immature Ixodes dammini (Acari: Ixodidae) following incremental removal of deer. J Med Entomol. 1993 1;30(1):144–50. 8433321 10.1093/jmedent/30.1.144

[34] Dolan MC, Maupin GO, Schneider BS, Denatale C, Hamon N, Cole C, et al Control of immature Ixodes scapularis (Acari: Ixodidae) on rodent reservoirs of Borrelia burgdorferi in a residential community of southeastern Connecticut. J Med Entomol. 2004 11;41(6):1043–54. 15605643 10.1603/0022-2585-41.6.1043

[35] Daniels TJ, Fish D, Schwartz I. Reduced abundance of Ixodes scapularis (Acari: Ixodidae) and Lyme disease risk by deer exclusion. J Med Entomol. 1993 11;30(6):1043–9. 8271246 10.1093/jmedent/30.6.1043

[36] Daniels TJ, Fish D, Falco RC. Evaluation of host-targeted acaricide for reducing risk of Lyme disease in southern New York state. J Med Entomol. 1991 7;28(4):537–43. 1941916 10.1093/jmedent/28.4.537

[37] Montibeller G. From (and to) a new generation of multi-criteria decision analysts: An introduction to the field and a personal view on its future In: E M, editor. The Operational Research Society Birmingham 2005 p. 17–30.

[38] Guitouni A, Martel J-M. Tentative guidelines to help choosing an appropriate MCDA method Eur J Oper Res. 1998;109(2):501–21.

[39] Zinsstag J, Schelling E, Wyss K, Mahamat MB. Potential of cooperation between human and animal health to strengthen health systems. Lancet. 2005 12 17;366(9503):2142–5. 16360795 10.1016/S0140-6736(05)67731-8

