# Supplementary material for: Adaptation and Evaluation of a Multi-Criteria Decision Analysis Model for Lyme Disease Prevention
Source: PLoS One. 2015 Aug 21;10(8):e0135171. doi: 10.1371/journal.pone.0135171 (PMC4546612; doi:10.1371/journal.pone.0135171)
Supplement: S1 Fig — (PDF) [file pone.0135171.s001.pdf]

S1 Fig. GAIA decision maps for (A) the 9 Swiss stakeholders (S1 to S9) considering all criteria, interventions and weighting schemes (Delta=95.1%, meaning that 95.1% of the information is conserved in the two-dimensional figure) and (B) the 8 Quebec stakeholders (S1 to S8) as computed in Aenishaenslin et Al. 2013 (16).

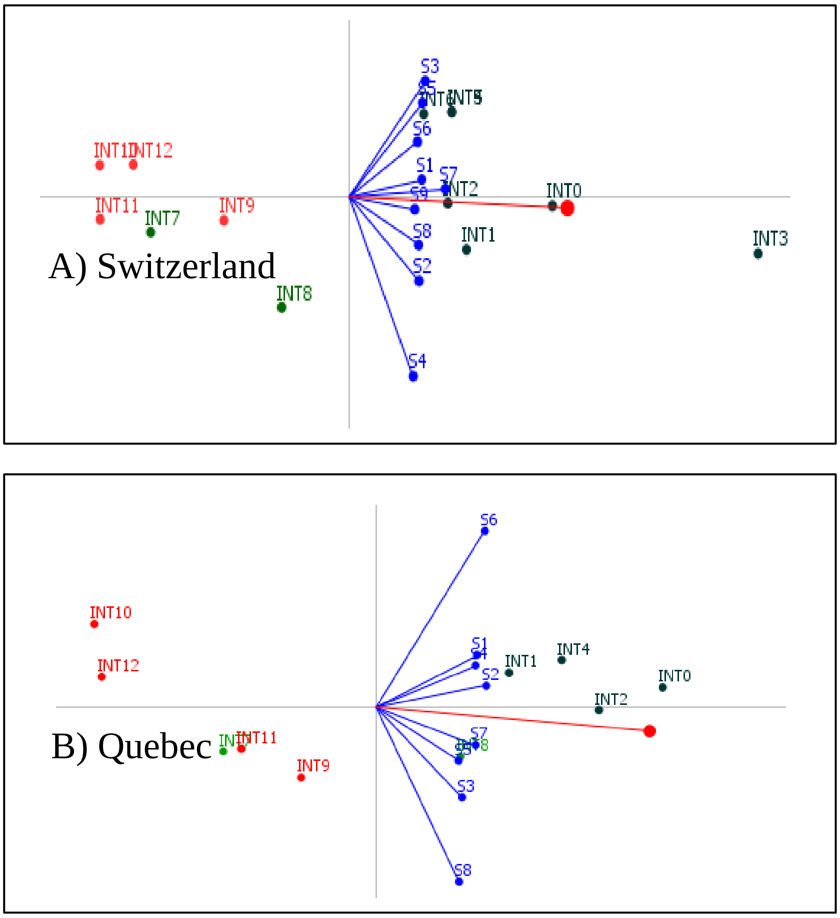

Legend: Interventions removed from the Swiss model have been removed from the map and all interventions were renamed to allow comparisons with the Swiss model. Delta=97.5%). Interventions in grey are those targeting human populations; in green are those targeting ticks directly in the environment; and in red are those targeting ticks thought actions on hosts (INT0 Status quo; INT1 Reduction of human visits to high-risk public areas via the use of fences or prohibitive signs; INT2 Human vaccination ; INT3 Large communication campaign; INT4 Making available special clinics for diagnosis of complex cases; INT5 Making available special clinics for complex LD cases management; INT6 Learning sessions for physicians; INT7 Small scale acaricide application; INT8 Small scale landscaping; INT9‘4-poster' device; INT10 Deer hunting; INT11 Exclusion of deer by fencing; INT12 ‘Damminix’ device)
