# Supplementary material for: Adaptation and Evaluation of a Multi-Criteria Decision Analysis Model for Lyme Disease Prevention
Source: PLoS One. 2015 Aug 21;10(8):e0135171. doi: 10.1371/journal.pone.0135171 (PMC4546612; doi:10.1371/journal.pone.0135171)
Supplement: S1 Table — (DOCX) [file pone.0135171.s002.docx]

**S1 Table. Measurement units for all selected criteria**

| **Criteria** | **Measurement units** |
| --- | --- |
|  |  |
| PHC1 Reduction in incidence of human cases | 0: Nil; 1: Low; 2: Moderate; 3: High |
| PHC2 Reduction in entomological risk | 0: Nil; 1: Low; 2: Moderate; 3: High |
| PHC3 Impacts of adverse health effects | 0: Nil; 1: Indirect effects on mental or social health; 2: Direct effects on physical health |
| PHC4 Reduction in incidence of disseminated LD human cases | 0: Nil; 1: Reduction of LD cases in general; 2: Reduction of disseminated cases specifically |
| AEC 1 Impact on habitat | Surface*Sensitivity*Intensity^1^  Surface : 1: Nil; 2: Small scale; 3: Large scale  Sensitivity: 1: Nil; 2: Land ; 3: Water ; 4: Land and water  Intensity: 1: Nil; 2: Fences; 3: Mowing; 4: Acaricides; 5: Removal of vegetation or burning |
| AEC 2 Impact on wildlife | Number*Species*Intensity^2^  Number : 1 : Nil; 2 : effect on specific species; 3 : Effect on several species  Species: 1: Nil. 2: low valued species; 3: Highly valued species  Intensity: 1: No effect; 2: Morbidity; 3: Mortality |
| SIC 1 Level of public acceptance | 1: Nil; 2: Low; 3: Moderate; 4: High |
| SIC 2 Proportion of population benefitting from intervention | 1 : <25%; 2 : 25-50%; 3 : 50-75%; 4 : >75% |
| SIC3 Level of public awareness | 0: Nil. The intervention targets the reduction in tick density; 1: Low. The intervention targets human populations but does not have the objective to raise the awareness; 2: Moderate. The intervention has the objective to raise awareness in a passive way; 3: High. The intervention has the objective to raise awareness in an active way. |
| SEC1 Cost to the public sector | 0: Nil; 1: Low; 2: Moderate; 3: High |
| SEC2 Cost to the private sector | 0: Nil; 1: Low; 2: Moderate; 3: High |
| SEC3 Delay before results | 1: Days; 2: Weeks; 3: Months; 4 : Years |
| SEC4 Complexity | 1: Simple (Minor institutional changes); 2:Intermediate (necessitates new hires); 3: Moderate Necessitates new work teams in one sector of intervention; 4: Complex (requires inter-sectoral/inter-institutional changes); 5: Very complex (necessitates creation of new structures or organisations) |
| SEC5 Impact on organisation’s credibility | 0: Nil; 1: Low; 2: Moderate; 3: High |
| SEC6 Sustainability of effect | 1: Low. The intervention must be re-applied for a long-term effect; 2: Moderate. The intervention must be maintained for a time before the appearance of an effect, but the effect lasts for a long time; 3: High. The effect lasts for a long time without the need to re-apply the intervention. |
| SEC7 Level of coherence with the European strategies | 0: Nil. The intervention is not recommended by European authorities; 1: the intervention is recommended by the European authorities. |
