# Supplementary material for: Adaptation and Evaluation of a Multi-Criteria Decision Analysis Model for Lyme Disease Prevention
Source: PLoS One. 2015 Aug 21;10(8):e0135171. doi: 10.1371/journal.pone.0135171 (PMC4546612; doi:10.1371/journal.pone.0135171)
Supplement: S2 Table — (DOCX) [file pone.0135171.s003.docx]

**S2 Table. Performance matrix of the model adapted for Switzerland**

|  | **PHC1** | **PHC2** | **PHC3** | **PHC4** | **AEC1** | **AEC2** | **SIC1** | **SIC2** | **SIC3** | **SEC1** | **SEC2** | **SEC3** | **SEC4** | **SEC5** | **SEC6** | **SEC7** |
| --- | --- | --- | --- | --- | --- | --- | --- | --- | --- | --- | --- | --- | --- | --- | --- | --- |
| **INT0** | 1 | 0 | 0 | 1 | 1 | 1 | 3 | 4 | 2 | 1 | 0 | 3 | 1 | 1 | 3 | 1 |
| **INT1** | 2 | 0 | 1 | 1 | 1 | 1 | 2 | 1 | 3 | 1 | 0 | 1 | 1 | 2 | 2 | 0 |
| **INT2** | 2 | 0 | 2 | 1 | 1 | 1 | 3 | 4 | 1 | 1 | 3 | 2 | 3 | 2 | 2 | 0 |
| **INT3** | 3 | 0 | 0 | 2 | 1 | 1 | 4 | 4 | 3 | 2 | 0 | 2 | 3 | 2 | 2 | 1 |
| **INT4** | 0 | 0 | 0 | 2 | 1 | 1 | 4 | 3 | 1 | 1 | 2 | 2 | 4 | 2 | 2 | 0 |
| **INT5** | 0 | 0 | 0 | 2 | 1 | 1 | 4 | 3 | 1 | 1 | 2 | 2 | 4 | 2 | 2 | 0 |
| **INT6** | 0 | 0 | 0 | 2 | 1 | 1 | 4 | 4 | 0 | 1 | 1 | 4 | 1 | 2 | 2 | 1 |
| **INT7** | 2 | 3 | 2 | 1 | 16 | 12 | 1 | 2 | 0 | 2 | 0 | 2 | 4 | 3 | 1 | 0 |
| **INT8** | 2 | 3 | 0 | 1 | 12 | 4 | 1 | 2 | 0 | 2 | 0 | 2 | 4 | 3 | 1 | 0 |
| **INT9** | 2 | 3 | 2 | 1 | 3 | 12 | 3 | 2 | 0 | 2 | 1 | 4 | 4 | 2 | 1 | 0 |
| **INT10** | 1 | 2 | 2 | 1 | 3 | 18 | 2 | 2 | 0 | 0 | 0 | 4 | 3 | 3 | 1 | 0 |
| **INT11** | 1 | 2 | 1 | 1 | 12 | 6 | 1 | 2 | 0 | 2 | 2 | 4 | 3 | 4 | 1 | 0 |
| **INT12** | 1 | 1 | 2 | 1 | 3 | 8 | 3 | 2 | 0 | 2 | 1 | 3 | 4 | 2 | 1 | 0 |

**Criteria list:**

PHC1: Reduction in incidence of human cases

PHC2: Reduction in entomological risk

PHC3: Impacts of adverse health effects

PHC4: Reduction in incidence of disseminated LD human cases

AEC1: Impact on habitat

AEC2: Impact on wildlife

SIC1: Level of public acceptance

SIC2: Proportion of population benefitting from intervention

SIC3: Level of public awareness

SEC1: Cost to the public sector

SEC2: Cost to the private sector

SEC3: Delay before results

SEC4 : Complexity

SEC5: Impact on organisation’s credibility

SEC6: Sustainability of effect

SEC7: Level of coherence with the European strategies

**Interventions list:**

INT0: Status quo

INT1: Reduction of human visits to high-risk public areas via the use of fences or prohibitive signs

INT2: Human vaccination

INT3: Large communication campaign

INT4: Making available special clinics for diagnosis of complex cases

INT5: Making available special clinics for complex LD cases management

INT6: Learning sessions for physicians

INT7: Small scale acaricide application

INT8: Small scale landscaping

INT9: ‘4-poster' device

INT10: Deer hunting

INT11: Exclusion of deer by fencing

INT12: ‘Damminix’ device
